# Successful human long-term application of *in situ* bone tissue engineering

**DOI:** 10.1111/jcmm.12296

**Published:** 2014-05-06

**Authors:** Raymund E Horch, Justus P Beier, Ulrich Kneser, Andreas Arkudas

**Affiliations:** a Department of Plastic and Hand Surgery, Laboratory for Tissue Engineering and Regenerative Medicine, University Hospital Erlangen, Friedrich Alexander University Erlangen-NuernbergErlangen, Germany; b Department of Hand, Plastic and Reconstructive Surgery, Burn Center, BG Trauma Center Ludwigshafen, University of HeidelbergHeidelberg, Germany

**Keywords:** tissue engineering, human application, long term success, large bone defect, arteriovenous loop, clinical translation, regenerative medicine

## Abstract

Tissue Engineering (TE) and Regenerative Medicine (RM) have gained much popularity because of the tremendous prospects for the care of patients with tissue and organ defects. To overcome the common problem of donor-site morbidity of standard autologous bone grafts, we successfully combined tissue engineering techniques for the first time with the arteriovenous loop model to generate vascularized large bone grafts. We present two cases of large bone defects after debridement of an osteomyelitis. One of the defects was localized in the radius and one in the tibia. For osseus reconstruction, arteriovenous loops were created as vascular axis, which were placed in the bony defects. In case 1, the bone generation was achieved using cancellous bone from the iliac crest and fibrin glue and in case 2 using a clinically approved β-tricalciumphosphate/hydroxyapatite (HA), fibrin glue and directly auto-transplanted bone marrow aspirate from the iliac crest. The following post-operative courses were uneventful. The final examinations took place after 36 and 72 months after the initial operations. Computer tomogrphy (CT), membrane resonance imaging (MRI) and doppler ultrasound revealed patent arterio-venous (AV) loops in the bone grafts as well as completely healed bone defects. The patients were pain-free with normal ranges of motion. This is the first study demonstrating successfully axially vascularized *in situ* tissue engineered bone generation in large bone defects in a clinical scenario using the arteriovenous loop model without creation of a significant donor-site defect utilizing TE and RM techniques in human patients with long-term stability.

## Introduction

Tissue defects in poorly vascularized recipient areas require the transplantation of replacement tissue that creates a donor-site defect elsewhere in the body. This has challenged the search for alternative means and has fostered the development of new scientific areas such as Tissue Engineering. Treatment of large bone defects after trauma, tumour or infection remains such a major challenge in different surgical fields such as orthopaedic surgery, neurosurgery, head and neck surgery or plastic surgery. Autologous bone grafting still represents the gold standard for osteogenic bone replacement, although availability is limited in quality and quantity [1]. Furthermore, autologous bone grafting is associated with a significant donor-site morbidity that increases with the amount of transplanted bone [2]. For large bone defects and cases of limited vascular supply of the host bed, only free microsurgically transplanted vascularized bone grafts such as fibula, scapula, iliac crest or others lead to optimal bone healing and survival of the bone graft [3,4].

A multitude of different synthetic bone substitutes have been developed for bone replacement to overcome the inherent shortcomings and donor-site morbidities of autologous bone grafting. Adequate vascularization plays a crucial role for transferring tissue engineering models from the *in vitro* to the *in vivo* environment as nutrition and oxygen supply *via* diffusion is limited to a maximum range of 200 μm [5]. The majority of the tissue engineering approaches uses the so-called ‘extrinsic’ mode of vascularization from the periphery, although this model is limited to small defects with high vascularization potential. Intrinsically vascularized tissue engineered constructs rely on a vascular axis and can be transferred to the defect site using microsurgical techniques. Terheyden *et al*. showed a clinical application of a custom-designed axially vascularized bone graft using the latissimus dorsi muscle as a vascular carrier leaving a significant muscle donor-site defect [6].

To overcome the problem of donor-site morbidity (leaving neither bone nor muscle defect), Erol and Spira developed the rat arteriovenous ‘arterio-venous (AV)’ loop model using only a vein graft as a vascular axis [7]. Over the past decade, we augmented this model for bone tissue engineering purposes. We were able to demonstrate axial vascularization of different bone matrices as well as bone generation [8,9]. We recently established the sheep AV loop model as a large animal model of axial vascularization [10]. We could also demonstrate axial vascularization of a clinically approved bone substitute with clinically relevant dimensions in the sheep, upscaling previous results from small animal experiments [11,12].

To verify this promising technique from bench to bedside in the human recipient, this is the first study demonstrating *de novo* bone generation in large bone defects in a clinical scenario using the arteriovenous loop model without creation of a significant donor-site defect in patients.

## Materials and methods

Between 2006 and 2009, two patients presented with large bone defects after debridement of an osteomyelitis. One of the defects was localized in the radius and one in the tibia. In all two cases, the osteomyelitis caused extensive poorly vascularized bony defects, which required vascularized bone grafts. To prevent any kind of donor-site morbidity but to achieve a vascularization inside the bone grafts in the poorly vascularized recipient areas, only a vein graft as a vascular axis was implanted into the bone graft using microsurgical techniques.

### Case 1

A 39-year-old patient sustained an open tibial fracture in 1991 in a motorcycle accident. A multitude of operative interventions resulted in an instable scar of the lower leg. The patient was referred to our department in November 2006 with a suspicion of an osteomyelitis of the tibia, which was noticed in membrane resonance imaging (MRI) after the patient hit the leg another time. An angiography of the left leg revealed that the lower leg was perfused only by collaterals. After surgical debridement and prolonged antibiotic treatment, the bony and soft tissue defects were reconstructed using an arteriovenous loop as a vascular connection for a free gracilis muscle flap transfer in one stage. Post-operatively the bypass showed an insufficient perfusion because of arteriovenous loop failure. After flap debridement and loop revision, a vacuum-assisted closure (VAC) therapy was applied temporarily and was followed by a second arterial bypass reconstruction and free rectus abdominis muscle flap with skin grafts. Initially, the post-operative course was then uneventful. However, 2 years post-operatively, the patient was readmitted with redness and swelling of the lower leg. She was found to suffer again from a severe osteomyelitis of the tibia. After multiple radical osseus and periosteum debridements and repeated application of VAC therapy, a bony defect of the tibia of 8 × 2.5 cm remained. As the patient refused another free flap reconstruction such as with a free vascularized tibia or free vascularized partial scapula despite the large bony defect, we discussed an attempt at minimally invasively transplanting cancellous bone chips augmented with an arteriovenous axis for osseus reconstruction. Therefore, an arteriovenous loop was created using a vein graft from the lower contralateral leg interposed between the popliteal artery (end-to-side microsurgical anastomosis) and the popliteal vein (end-to-side) penetrating the tibial bone defect. The bone defect was then filled with a custom-made bone substitute using small cancellous bone chips from the iliac crest and fibrin glue (Tisseel^©^, Baxter, Vienna, Austria). Finally, the moulded mass in the defect was covered and held together using a vicryl mesh with a corresponding hole for the vascular pedicle for the arterial and the venous side each.

### Case 2

**Fig. 1:**
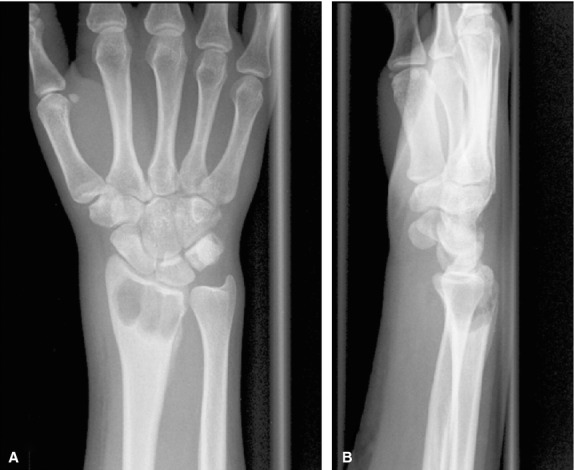
Pre-operative X-ray of the wrist (case 2) showing cystic formations at the distal radius. (**A**) Posteroanterior radiograph. (**B**) Lateral radiograph.

**Fig. 2:**
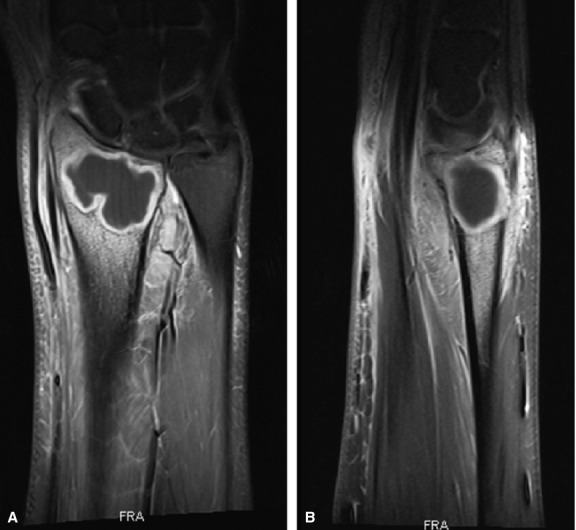
Pre-operative MRI of the lower arm (case 2) showing cystic formations at the distal radius consistent with a giant cell carcinoma. (**A**) Coronar reconstruction. (**B**) Sagittal reconstruction.

In December 2009, a 20-year-old woman was referred to us with a history of repetitive wrist pain without any trauma. On admission, plain radiographs and MRI of the lower arm and wrist showed cystic formations at the distal radius morphologically consistent with a suspected giant cell carcinoma (Figs 1 and 2). At operative intervention, an abscess formation was detected in the distal radius and taken out leaving a bone defect of 3 × 4 cm. Histological examination showed no malignancy and no giant cells but an osteomyelitis.

**Fig. 3:**
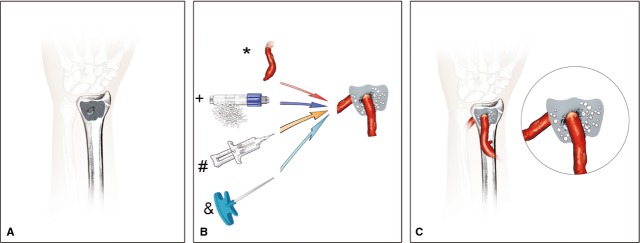
Preparation of the bone construct (case 2): After creation of the bone defect (**A**) the bone construct was composed of a clinically approved β-tricalciumphosphate/hydroxyapatite matrix (+), fibrin glue (#) and directly auto-transplanted bone marrow aspirate from the right iliac crest (&) (**B**). An arteriovenous loop was created by a vein graft (*) from the lower arm interposed between the radial artery on the volar site (end-to-side microsurgical anastomosis) and the cephalic vein (end-to-end) at the dorsal site of the lower arm penetrating the bone construct (**C**).

To minimize donor-site morbidity (neither bone nor muscle defect), the patient was selected for the generation of an axially vascularized tissue engineered bone construct. The vascularization was achieved using an arteriovenous loop created by a vein graft from the lower arm interposed between the radial artery on the volar site (end-to-side microsurgical anastomosis) and the cephalic vein (end-to-end) at the dorsal site of the lower arm penetrating the bone construct. The bone construct was composed of a clinically approved β-tricalciumphosphate/HA (TRICOS^©^, Baxter Healthcare S.A.), fibrin glue (Tisseel^©^, Baxter) and directly auto-transplanted bone marrow aspirate from the right iliac crest (Fig. 3). Intraoperative a VAC therapy at the dorsal site of the lower arm was applied because of the soft tissue swelling, which could be removed after 13 days before successful surgical closure.

## Results

### Case 1

Post-operatively, no signs of infection occurred. Radiographs and computer tomogrphy (CT) imaging revealed that the tibial bone defect was healed without any signs of osteomyelitis. The final review took place 2 years after the bone reconstruction. The patient remained pain-free and started water skiing until now, which is 5 ½ years after this treatment.

### Case 2

**Fig. 4:**
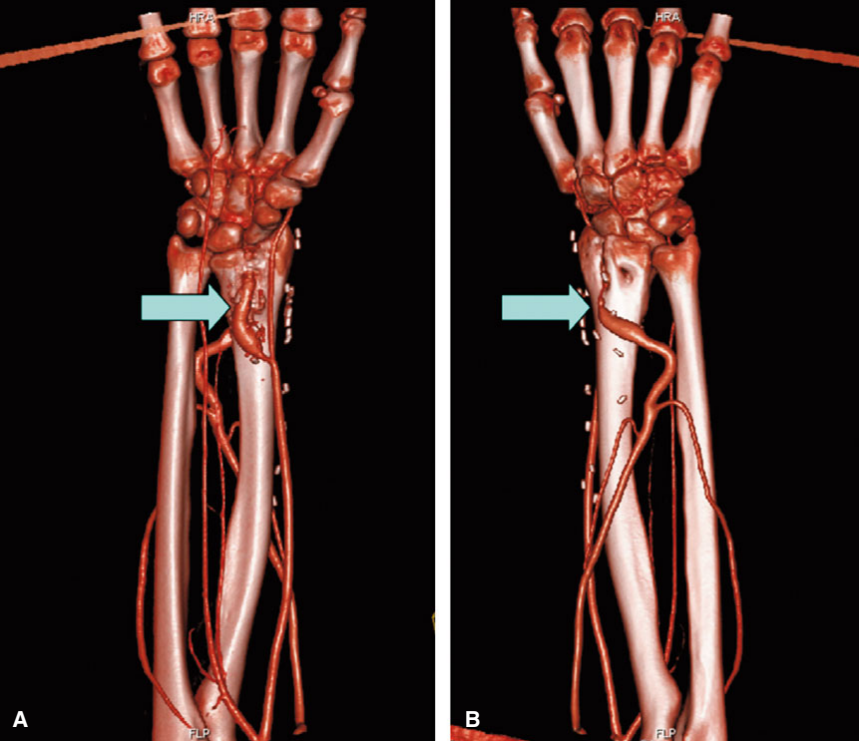
Magnetic resonance angiography 14-month post-operative (case 2) showing a patent arteriovenous loop as well as a completely healed bone defect in the radius. (**A**) Palmar view. (**B**) Dorsal view. Arrows: arteriovenous loop.

**Fig. 5:**
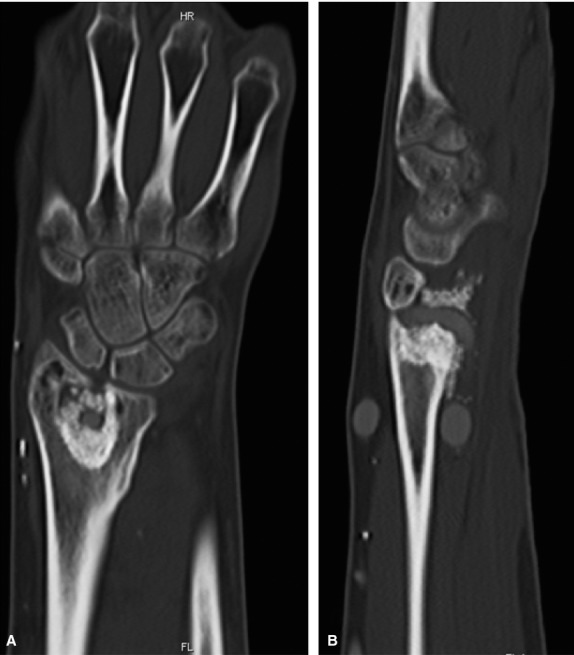
CT scan 14-month post-operative (case 2) demonstrating a completely healed bone defect in the distal radius. (**A**) Coronar reconstruction. (**B**) Sagittal reconstruction.

**Fig. 6:**
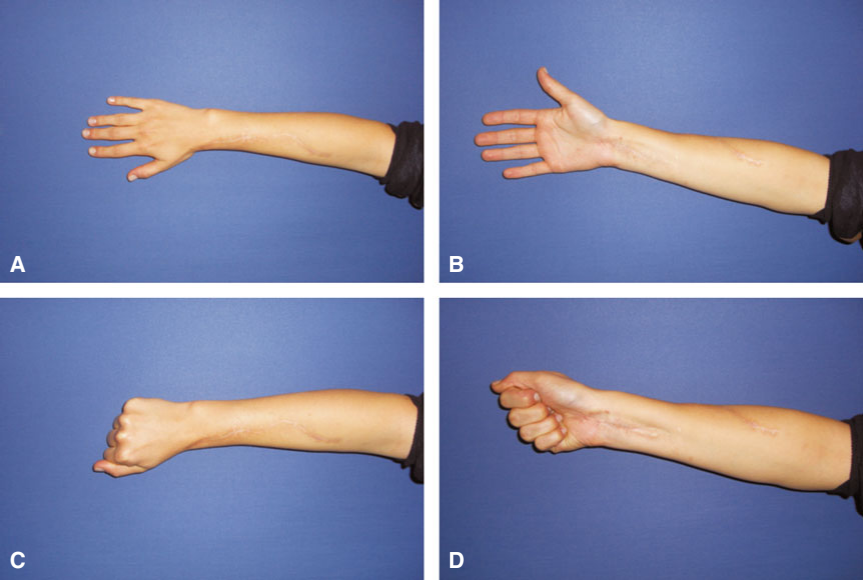
14 months after bony reconstruction, patient showed full range of motion in the hand (case 2).

The following post-operative course was uneventful. Patency of the AV loop was verified using doppler ultrasound. In the follow-up examination, the range of active and passive motion was normal and the patient was pain-free. At 14 and at 32 months follow-up, CT and MRI revealed a patent arteriovenous loop as well as a completely healed bone defect in the radius (Figs 4–6). She is subjectively free of symptoms until now.

## Discussion

Until now, Tissue Engineering techniques have frequently been shown to be promising *in vitro* and *in vivo* in experimental settings, but have widely failed to enter the clinical routine when it comes to large defects or major organ functional replacements [13]. One of the key roles for this insufficient transition into clinical practice has been discussed to be dependent on the lack of sufficient vasculature at the time of transplanting laboratory-grown constructs into relevant and especially into poorly vascularized recipient areas. Large bone defects present a prototype of such difficult to handle clinical replacement problems, as vascularized bone grafts are associated with a significant donor-site morbidity and non-vascularized bone grafts do not heal into problematic sites. The optimal bone graft for any successful reconstruction of a large osseus defect would consist of a custom-designed vascularized bone substitute without creating any donor-site morbidity. Almost all bone tissue engineering approaches generate bone either *in vitro* or *in vivo* using an extrinsic vascularization in a small animal model [14]. Over the last years, a lot of these published studies report of successful bone tissue engineering without taking the next step towards clinical application [15,16]. This is because of a missing vascular model, which is mandatory when implantation in areas of limited perfusion and impaired regenerative capacity is intended. Reconstructive surgeons aim to generate axially vascularized bone grafts, which allow transplantation to the defect site using microsurgical anastomosis [17]. Autologous vascularized bone grafts can be obtained from different locations such as fibula, scapula, iliac crest and others to accomplish adequate reconstruction [2–4]. Although this technique still represents the gold standard for large osseus defects, it is associated with several shortcomings and complications, including limited quantities of bone for harvest, often resulting in significant donor-site morbidity [2]. Furthermore, autologous bone grafts can be adapted to the required shape only to a certain extent. Using pre-fabrication and pre-lamination techniques, axially vascularized tissue grafts can be generated prior to the transplantation. These techniques were first described by Shen in 1982 and further augmented by Pribaz *et al*. [18,19]. Originally, these techniques were created to axially vascularize existing tissues and not to grow new tissues. By combining the pre-fabrication technique and tissue engineering approaches, *de novo* axially vascularized tissue formation was achieved. Pre-fabricated bone grafts can be generated using two different methods: Either a bone graft is transplanted in an intrinsically vascularized tissue (skin, muscle, *etc*.) and after the graft is vascularized in an extrinsic pathway by the donor tissue, the whole construct can be transferred to the recipient site using the vascular pedicle of the host tissue. This technique has already been applied in a clinical setting by Terheyden *et al*. in 2004 [6]. They were able to build a custom-shaped mandibular bone graft using the latissimus dorsi muscle as a vascular carrier. Although a large donor bone defect could be prevented using this method, there was still a major donor-site morbidity in terms of latissimus dorsi muscle that had to be harvested. However, this initially promising reconstruction with a still significant donor-site defect failed to show long-term stability and had to be removed [20]. To prevent any kind of significant donor-site morbidity, it seems a feasible new possibility to only harvest a vessel as a vascular axis that then can be implanted into the bone graft using microsurgical techniques and after a pre-vascularization time, the bone graft can be transferred using the implanted vascular pedicle. An animal model of this method is the arteriovenous loop model, which was first described by Erol and Spira in 1979 in the rat [7]. Here, a venous graft was interposed between the femoral artery and vein in the thigh to create an arteriovenous loop as a vascular carrier. This vascular axis was used to vascularize a defined tissue volume, which was placed in an isolation chamber around the AV loop [21,22]. Because this technique may well overcome the known problems of sufficient initial vascularization in tissue engineering, our group has focussed on tissue engineering of axially vascularized bone grafts by further developing this rat model over the last decade. We were able to show successful vascularization of different bone matrices including the clinically approved HA/TCP granula matrix of case 2 [23,9]. Furthermore, we used fibrin as a release system for angiogenic growth factors such as VEGF and bFGF to accelerate the process of vascularization [24,25]. Based on our findings, we developed the sheep arteriovenous loop model as an essential step towards clinical application [10,26]. We were able to axially vascularize bone matrices with clinically relevant dimensions using the arterio-venous-loop (AVL) within 6 weeks after implantation [11]. Recently, we showed successful enhanced vascularization of bone matrices using chambers with incorporated pores to allow additional extrinsic vascularization of matrices. We were able to show that the intrinsic pathway of the AV loop and the extrinsic vascularization connect after a pre-vascularization period allowing transplantation of the entire construct using the vascular pedicle. Already at 2 weeks, constructs begin to vascularize from the periphery and the AVL. On the basis of our findings, we used a vicryl mesh as a cover and fixation of the individually moulded bone construct in case 1 and fibrin gel only in case 2 to accelerate vascularization of the constructs as a prerequisite for any cell-based study. Furthermore, bone defects were filled with custom-shaped constructs, which were moulded to fit perfectly into the defects. Using this approach, we were able to perform a single-stage AVL implantation and matrix application containing osteogenic cells in the defect site in the presented cases. In both cases, we used a venous graft interposed between an artery and vein as the vascular axis in the bone graft. The interpositional venous graft underlies subsequent arterialization and possesses the capacity of inducing angiogenesis as shown by previous experiments [27]. This is a prerequisite for clinical application because standard vein bypass techniques can be used and with increasing length of the vascular axis, donor-site morbidity becomes an issue. In case 1, we used the saphena parva vein of the contralateral leg, and in case 2, a subcutaneous vein of the same lower arm, so donor-site morbidity was not prevalent.

Beside vascularization of bone grafts, bioartificial bone substitutes consist of mainly three more components, which should be addressed in any successful tissue engineering strategy: (*i*) extracellular matrix; (*ii*) osteogenic cells; and (*iii*) growth factors. A vast variety of different scaffolds have been described for bone tissue engineering applications such as HA and β-tricalcium phosphate (TCP) or processed bovine cancellous bone [28,29]. In case 1, we used cancellous bone from the iliac crest as a cell and matrix source leaving a negligible donor-site morbidity. Calcium phosphate scaffolds, such as HA and TCP, are osteoconductive and osteoinductive ceramics and already widely used in clinical applications [30]. Hence, we previously showed in the small and large AVL animal model successful vascularization of the clinically approved HA/TCP granula matrix [9,12]. Conclusions deductible from these studies were transferred in the clinical setting. Therefore, we replaced the cancellous bone matrix of case 1 with the HA/TCP matrix in case 2, thereby eliminating the possible complications associated with the harvest of cancellous bone from the iliac crest. In both cases, we used fibrin as a binding agent for the bone matrix. Furthermore, fibrin offers several other advantages as a matrix component. Fibrin is known as a release system for growth factors and as a cell carrier [31,32]. Although we used aprotinin in a concentration of 1500 IU/ml to slow down fibrinolysis after implantation, we were able to show in previous AVL studies that fibrin gel will degrade and been replaced by connective vascularized tissue within several weeks.

Osteogenic cells, either primarily implanted or attracted by osteogenic growth factors, are essential for any bone tissue engineering strategy [33]. In both cases, we used bone marrow as a natural reservoir of different osteogenic cells like mesenchymal stem cells (MSCs), bone marrow stromal cells, osteoblasts and osteoclasts. Although it is known that bone morphogenic proteins (BMPs) can generate bone by attracting osteogenic cells, in the future, BMP stimulation might become dispensable. We were able to show in the AVL sheep model that directly auto-transplanted MSCs lead to a *de novo* bone formation comparable to application of BMP alone or in combination with expanded MSCs [12]. Hence, we abandoned to apply additional BMPs as bone marrow contains beside of osteogenic cells, inter alia, different kinds of osteogenic and angiogenic growth factors.

In the long-term follow-up examination, over 3 1/2-year post-operatively patent arteriovenous loops were detectable as well as a completely healed bone defects showing successful osseus reconstruction. The patients are performing daily tasks and sporty activities proving stable bony reconstructions without any osteosynthetic fixation. In cases of irradiated or traumatized defect sites perhaps a two-stage procedure would meet the demands of a vascularized bone graft more appropriately. In these cases, the construct would be first implanted into a site of high vascularization potential (*e.g*. groin) with subsequent microsurgical transfer to the distant defect site using the vascular pedicle.

This is the first clinical study demonstrating axially vascularized *in situ* tissue engineered bone generation in large bone defects in a clinical scenario using the arteriovenous loop model without the creation of a significant donor-site defect. This technique will potentially expand the frontiers of reconstructive surgery towards custom-designed vascularized bone grafting without donor-site morbidity.
